# Elucidating Tricin-Lignin Structures: Assigning Correlations in HSQC Spectra of Monocot Lignins

**DOI:** 10.3390/polym10080916

**Published:** 2018-08-15

**Authors:** Wu Lan, Fengxia Yue, Jorge Rencoret, José Carlos del Río, Wout Boerjan, Fachuang Lu, John Ralph

**Affiliations:** 1 State Key Laboratory of Pulp and Paper Engineering, South China University of Technology, Guangzhou 510630, China; wu.lan@epfl.ch (W.L.); yuefx@scut.edu.cn (F.Y.); 2 DOE Great Lakes Bioenergy Research Center, Wisconsin Energy Institute, University of Wisconsin, Madison, WI 53706, USA; jralph@wisc.edu; 3 Department of Biological System Engineering, University of Wisconsin, Madison, WI 53706, USA; 4 Department of Biochemistry, University of Wisconsin, Madison, WI 53706, USA; 5 Instituto de Recursos Naturales y Agrobiologia de Sevilla (IRNAS), CSIC, 003495 Seville, Spain; jrencoret@irnase.csic.es (J.R.); delrio@irnase.csic.es (J.C.d.R.); 6 Department of Plant Systems Biology, VIB, 9052 Ghent, Belgium; woboe@psb.ugent.be; 7 Department of Plant Biotechnology and Bioinformatics, Ghent University, 9052 Ghent, Belgium

**Keywords:** tricin, lignin, model compound, HSQC, nuclear magnetic resonance (NMR), monocot

## Abstract

Tricin [5,7-dihydroxy-2-(4-hydroxy-3,5-dimethoxyphenyl)-4H-chromen-4-one] is a flavone that has been found to be incorporated in grass lignin polymers via 4′–O–β coupling. Herein, we investigated the tricin-lignin structure using nuclear magnetic resonance (NMR) methods by comparing the 1H–13C heteronuclear correlation (HSQC) NMR spectra of the isolated lignin with a series of dimeric and trimeric tricin-4′–O–β-ether model compounds. Results showed that the tricin moiety significantly affects the chemical shift of the Cβ/Hβ of 4′–O–β unit, producing peaks at around δC/δH 82.5–83.5/4.15–4.45, that differ from the Cβ/Hβ correlations from normal 4–O–β units formed solely by monolignols, and that have to date been unassigned.

## 1. Introduction

Lignin, a phenylpropanoid polymer, is one of the major components of plant cell walls. Many aspects of lignin structure and biosynthesis remain elusive [1]. Several novel monomers and the details of various interunit linkages in lignin polymers were not discovered until rather recently [2,3,4]. For example, tricin [5,7-dihydroxy-2-(4-hydroxy-3,5-dimethoxyphenyl)-4H-chromen-4-one], a flavone that derived from a combination of the shikimate and acetate/malonate-derived polyketide pathways, was only recently revealed to be present in the lignin polymer from wheat straw according to the characteristic correlations in the 1H–13C heteronuclear correlation (HSQC) spectrum [5]. Follow-up studies using biomimetic radical coupling reactions authenticated tricin as a monomer incorporated in polymeric lignin via 4′–O–β-coupling with monolignols [6]. Metabolite profiling of the lignifying tissue of maize elucidated the incorporation pathway of tricin into lignin [7], and the absolute contents of tricin in the lignin from various plant species were also investigated using thioacidolysis and liquid chromatography–mass spectrometry (LC–MS) [8].

HSQC is the most frequently applied 2D nuclear magnetic resonance (NMR) technique for lignin characterization because it provides comprehensive information on the types of units and their characteristic interunit linkages in the polymer. Signal assignments in a HSQC spectrum of lignin are mostly based on the assignments of low molecular weight model compounds, particularly dimers and trimers, which provide the key NMR data for structural authentication. In this short communication, we report on work in which we synthesized several 4′–O–β-coupling products of tricin with coniferyl and sinapyl alcohol, as well as a trimer that resulted from further cross-coupling with another monolignol. By comparing the NMR data of the model compounds with those of an isolated lignin sample, we have elucidated the tricin-lignin structure (especially the sidechain structure) in polymeric lignin and reveal characteristic new correlations in the HSQC spectrum.

## 2. Materials and Methods

### 2.1. Materials

All chemicals and solvents used in this study were purchased from commercial sources (analytical grade) and used without further purification. The wheat sample was the same as that used in a previous publication [5]; chalcone synthase (CHS)-deficient and control maize samples were those used in another earlier study [9]. Thin-layer chromatography (TLC) plates (20 × 20 cm^2^, 1 mm, normal phase, Analtech. (Newark, NJ, USA) were used for raw product fractionation and purification using hexane/ethyl acetate or methanol/dichloromethane as eluent. Flash chromatography was performed using Biotage SNAP silica cartridges on an Isolera One instrument (Biotage, Uppsala, Sweden) using a hexane/ethyl acetate (EtOAc) gradient as eluent.

### 2.2. Syntheses of Model Compounds

Compound **1** Tricin-(4′–O–β)-coniferyl alcohol, compound **2** tricin-(4′–O–β)-syringyl alcohol, and compound **4** tricin-(4′–O–β)-syringyl alcohol-(4–O–β″)-coniferyl alcohol were all synthesized according to a previous study [6]. Compound **3** tricin-(4′–O–β)-coniferyl alcohol (4-O-methylated) was synthesized using the same method as other tricin-containing products, but using 3,4-dimethoxyacetophenone instead of 3-methoxy-4-hydroxyacetophenone as starting materials. All of the synthetic compounds were characterized by NMR and the data match those in a previous publication [6].

### 2.3. Acetylation of Model Compounds and Lignin

The model compound (10 mg) or enzyme lignin (25 mg) was dissolved in 0.25 mL pyridine and 0.1 mL acetic anhydride (1 mL pyridine and 0.5 mL acetic anhydride for lignin) and stirred for 2 h (12 h for lignin) at room temperature. When the reaction was completed, the solvent was evaporated under reduced pressure at 45 °C. Ethanol was added as co-solvent and repeatedly evaporated to completely eliminate the residue of pyridine and acetic anhydride. The acetylated samples can be readily dissolved in CDCl_3_ and then transferred to an NMR tube for characterization.

### 2.4. Nuclear Magnetic Resonance (NMR) Characterization

The model compound or lignin sample (10–25 mg) was dissolved in 0.7 mL of deuterated solvent (non-acetylated samples in DMSO-d6/pyridine-d5 4:1 *v/v*, and acetylated samples in CDCl_3_) and the solution was then transferred to an NMR tube for NMR acquisition. NMR spectra were recorded on a Bruker Biospin AVANCE 500 or 700 MHz spectrometer (Bruker, MA, USA) fitted with a cryogenically cooled 5-mm TCI (500 MHz) or quadruple-resonance 1H/31P/13C/15N QCI gradient cryoprobe (700 MHz) gradient probe with inverse geometry (proton coil closest to the sample). Bruker’s Topspin 3.1 (Mac) software was used to process spectra. The central solvent peaks were used as internal references (δC/δH CDCl_3_ 77.0/7.26; DMSO-d6 39.5/2.49).

## 3. Results and Discussion

The β-ether unit with its β–O–4-ether interunit linkage is the most abundant in lignin polymers and has already been studied comprehensively. It is well established that the methoxylation degree of the aromatic ring in β–O–4 aryl ethers affects the chemical shift of the Cβ/Hβ correlation in HSQC spectra, resulting in resolvable correlations between β–O–4-guaiacyl units and β–O–4-syringyl units. Recently, tricin was found to be incorporated into lignin via 4′–O–β-coupling with monolignols. Tricin contains one more carbon-carbon double bond and a carbonyl group conjugated to the 3′,5′-dimethoxylated aromatic ring in its structure, which is different from the β–O–4 interunit formed by the three canonical monolignols. Such an electronically different structure attached at the β-position of a monolignol might be expected to affect the chemical shifts of the Cβ/Hβ correlation.

**Figure 1:**
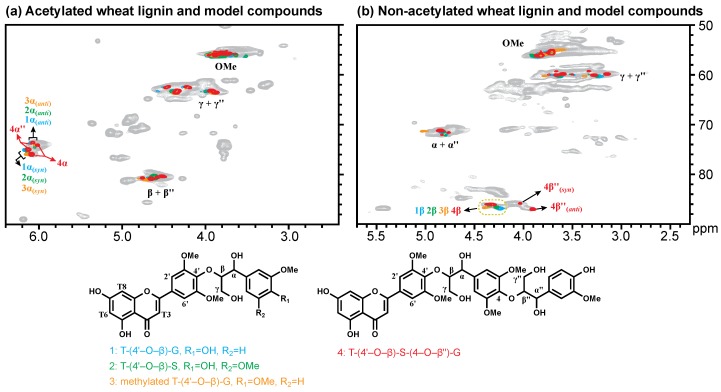
1H–13C heteronuclear correlation (HSQC) spectra of acetylated (**a**) and non-acetylated (**b**) milled wood lignin of wheat straw overlaid with the nuclear magnetic resonance (NMR) data from four tricin-containing model compounds.

To confirm our hypothesis, we synthesized four model compounds, including 3 dimers that result from tricin cross-coupled with coniferyl alcohol, sinapyl alcohol, and the phenol-etherified product represented by 4-O–methylated coniferyl alcohol and 1 trimer of tricin-(4′–O–β)-sinapyl alcohol-(4–O–β″)-coniferyl alcohol. By comparing the lignin HSQC spectra with those of the model compounds, we were able to resolve the tricin-lignin structure from these Cβ/Hβ correlations. The aromatic region of the tricin HSQC spectrum has been well studied previously [5,6], showing four characteristic correlations corresponding to C6/H6, C8/H8, C3/H3, and C2′6′/H2′6′ in tricin. Herein we focus on the sidechain structure of tricin-lignin from the “lignin side”. Figure 1 shows the sidechain region of the HSQC spectrum of wheat straw lignin overlaid with those of model compounds. For acetylated samples (Figure 1a), the signals of Cβ/Hβ and Cγ/Hγ (from tricin-monolignol interunit) fell into the same region as the signals of Cβ″/Hβ″ and Cγ″/Hγ″ (from monolignol-monolignol interunit). The correlations of Cα/Hα and Cα″/Hα″ in the two isomers of acetylated T-(4′–O–β)-S-(4–O–β″)-G were resolvable. In the case of non-acetylated samples (Figure 1b), the Cα/Hα and Cγ/Hγ correlations overlapped with the Cα″/Hα″ and Cγ″/Hγ″ and cannot be distinguished from each other. However, the chemical shifts of Cβ/Hβ in the tricin-(4′–O–β)-monolignol interunit were quite different from those in normal β–O–4 units formed by monolignols. The Cβ/Hβ correlations in the range of δC/δH 81.0–82.5/4.15–4.40 originated from the monolignol-(β–O–4)-guaiacyl units, whereas the peaks in the range of δC/δH 82.5–84.0/3.9–4.2 corresponded to the monolignol-(β–O–4)-syringyl units with syn-isomers upfield and anti-isomers downfield in the 1H dimension. However, the Cβ/Hβ peaks from tricin-(4′–O–β)-monolignol units located at around δC/δH 82.5–83.5/4.15–4.45 were sufficiently significantly displaced to be differentiated from the above two cases (Table 1). In fact, in the supplementary information (Supplementary Figure S1) from the original paper identifying tricin in wheat straw lignin [5], there is also a clear long-range (HMBC) correlation to this correlation peak, further supporting the assignment here.

The HSQC spectra of the lignin preparations from monocot species such as wheat [5,10,11], barley [12], sugarcane [13], elephant grass [14], *Brachypodium* (unpublished data in our lab), and oat (unpublished data in our lab) all clearly showed the correlations in the range of δC/δH 82.5–83.5/4.15–4.45, but researchers were not able to assign this correlation at the time because of the lack of data from model compounds. The lignin from coconut coir fibers contains tricin as well, but the content was too low to show the characteristic tricin correlations in the HSQC spectrum [8,15].

**Figure 2:**
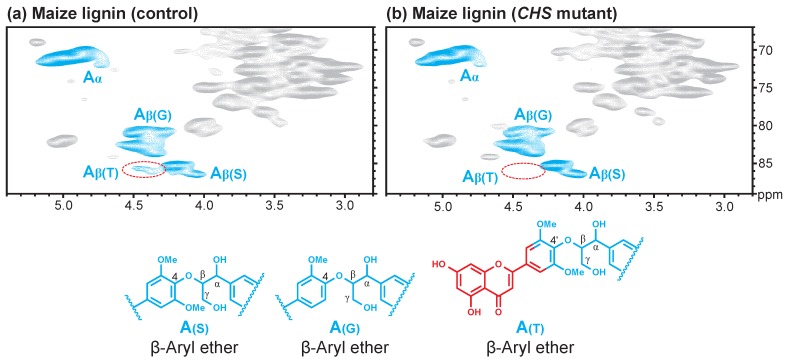
HSQC spectra of enzymatic lignin from control (**a**) and chalcone synthase (CHS) mutant (**b**) maize leaf.

To further confirm our hypothesis, we characterized the lignin isolated from the CHS-deficient maize and its wild-type control. CHS is the main enzyme that controls the carbon flux from the common pathway intermediate *p*-coumaroyl-CoA toward flavonoid biosynthesis. The tricin moiety was completely depleted in the CHS mutant and, therefore, the corresponding HSQC spectrum did not show the characteristic correlations from tricin in the aromatic region [9]. As was not appreciated at the time, however, the tricin-related Cβ/Hβ correlation in the lignin sidechain region was also depleted (Figure 2); the HSQC spectrum of the lignin isolated from wild-type samples clearly shows the Cβ/Hβ peak from tricin-(4′–O–β)-substructures (Figure 2).

## 4. Conclusions

In summary, the data from model compounds, together with the study of the lignin structure of different species, as well as the lignin from CHS mutant maize leaf and its corresponding wild-type control, unambiguously support our hypothesis that the tricin moiety affects the Cβ/Hβ 4′–O–β unit chemical shifts such that correlations in the region of δC/δH 82.5–83.5/4.15–4.45 in the HSQC spectrum are diagnostically assigned to these tricin-lignin unit Cβ/Hβ entities. As such, these correlations provide another “marker” for tricin in lignins, this time from the viewpoint of the lignin rather than the tricin moiety.

## Figures and Tables

**Table 1:** Chemical shift of the Cβ/Hβ and Cβ″/Hβ″ of the non-acetylated model compounds.

Title 1	Cβ/Hβ	Cβ″/Hβ″
1. T-(4′–O–β)-G	86.94/4.24	-
	86.38/4.34	
2. T-(4′–O–β)-S	86.63/4.27	-
	86.36/4.36	
3. 4–O-methylated T-(4′–O–β)-G	86.74/4.39	-
	86.21/4.49	
4. T-(4′–O–β)-S-(4–O–β″)-G	86.11/4.31	87.01/3.91
	86.04/4.38	85.85/4.03
